# Supplementary material for: Cost of implementing a doxycycline test-and-treat strategy for onchocerciasis elimination among settled and semi-nomadic groups in Cameroon
Source: PLoS Negl Trop Dis. 2023 Oct 18;17(10):e0011670. doi: 10.1371/journal.pntd.0011670 (PMC10615284; doi:10.1371/journal.pntd.0011670)
Supplement: S1 Table — Different measurement and cost valuation methodologies were used as the level of necessary detail was not obtained to accurately estimate the cost of several activities. S1 Table presents the methodology used to measure the cost of each activity, as well as the source of information for its valuation. (PDF) [file pntd.0011670.s001.pdf]

Table S1 – Methodologies by activity and sources used

Activities	Methodology	Source
Overheads	Mark-up	Partners' overhead percentage
Planning and coordination	Top-down gross-costing	Gross amount paid for this activity, sub-activity breakdown based on budget and implementer discussion
Advocacy	Top-down gross-costing	Gross amount paid for this activity, sub-activity breakdown based on budget and implementer discussion
Training	Top-down gross-costing	Gross amount paid for this activity, sub-activity breakdown based on budget and implementer discussion
Census	Bottom-up micro-costing	Pilot financial data and output data
Testing	Bottom-up micro-costing	Pilot financial data and output data
PCR analysis	Top-down micro-costing	Pilot financial data and output data
Treatment	Bottom-up micro-costing	Pilot financial data and output data
Monitoring of T&T	Bottom-up micro-costing	Pilot financial data and output data
