# Supplementary material for: Cost of implementing a doxycycline test-and-treat strategy for onchocerciasis elimination among settled and semi-nomadic groups in Cameroon
Source: PLoS Negl Trop Dis. 2023 Oct 18;17(10):e0011670. doi: 10.1371/journal.pntd.0011670 (PMC10615284; doi:10.1371/journal.pntd.0011670)
Supplement: S2 Table — Table of activities included in the costing work with detailed information on expenditures required for the completion of the related implementation activity. (PDF) [file pntd.0011670.s002.pdf]

Table S2: Table of activities included in standard cost

Activities	Included
Overheads	Mark-up, assumed 15% of direct costs
Planning and coordination	• Salaries, per diem and other personnel expenditures related to operational, management and coordination activities<br>• Supplies and other equipment attributable
Advocacy	Expenditures directly attributable to advocacy meetings activities:<br>• Salaries, per diem and other personnel expenditures related to operational, management and coordination activities<br>• Per diem and transportation expenses of attendees<br>• Material and supplies
Training	Expenditure related to training activities:<br>• Trainer and trainee per diem, accommodation, transportation, etc.<br>• Venue rental<br>• Material and supplies
Camp verification	Expenditure related to camp's occupancy verification:<br>• Purchase of satellite maps<br>• Development of application for optimisation of verification teams' itineraries<br>• Coordinator, MoH staff, CDDs, drivers, per diem, accommodation, transportation, etc.<br>• Material and supplies
Census	Expenditure related to community meetings, satellite maps verification and census of semi-nomadic population activities:<br>• Coordinator, MoH staff, CDDs, drivers, per diem, accommodation, transportation, etc.<br>• Material and supplies
Testing	Expenditure related to testing, skin snip collection, skin biopsies, and recording activities:<br>• Partner staff, CDDs, drivers, per diem, accommodation, transportation, etc.<br>• Material and supplies

PCR analysis	Expenditure related to packing, shipping, and testing negative skin snips activities:<br>• Bench fees for PCR tests<br>• Material and supplies<br>• Shipping fees
Treatment	Expenditure related to treatment distribution and monitoring activities:<br>• Partner staff, CDDs, per diem, accommodation, transportation, etc.<br>• Material and supplies (including Doxycycline, and patients' meal)
Monitoring of T&T	Expenditure related to Ministry of Health supervision activities:<br>• MoH staff, per diem, accommodation, transportation, etc.
