# Supplementary material for: Cost of implementing a doxycycline test-and-treat strategy for onchocerciasis elimination among settled and semi-nomadic groups in Cameroon
Source: PLoS Negl Trop Dis. 2023 Oct 18;17(10):e0011670. doi: 10.1371/journal.pntd.0011670 (PMC10615284; doi:10.1371/journal.pntd.0011670)
Supplement: Attachment — Submitted filename: PNTD-D-23-00377_reviewer - 22nd May, 2023.docx [file pntd.0011670.s004.docx]

PLOS Neglected Tropical Diseases

Cost of implementing doxycycline test-and-treat strategy for onchocerciasis elimination among settled and semi-nomadic groups in Cameroon

--Manuscript Draft--

| Manuscript Number: | PNTD-D-23-00377 |
| --- | --- |
| Full Title: | Cost of implementing doxycycline test-and-treat strategy for onchocerciasis elimination among settled and semi-nomadic groups in Cameroon |
| Short Title: | Cost of test-and-treat strategy for onchocerciasis elimination in Cameroon |
| Article Type: | Research Article |
| Keywords: | Doxycycline, Rivers; Onchocerciasis; Larvae; Parasitology; Public health; Economics; Costing |
| Abstract: | Background: Onchocerciasis is a neglected tropical disease with 217.5 million people globally ar e at risk of having the infection. Sightsavers carried out test-and-treat with doxycycline in settled and semi-nomadic communities of Massangam Health District in Cameroon. This paper presents the related cost of activity implementation in two community contexts, settled and semi-nomadic.  Methods: A mix of gross or micro-costing was used to identify cost components, and bottom-up and top-down approaches for their valuation. The opportunity costs of vehicle and equipment use was estimated and included. However, opportunity costs of building use and Ministry of Public Health staff salaries were not included. We only captured the incremental costs of implementing test and treatment activities as part of a functional annual community-directed treatment with ivermectin programme.  Results: We estimate an economic cost per person tested and cost per person treated in Massangam to be US$134 and US$664, respectively. Total implementation cost in the settled community was US$78,965 and US$69,677 in the semi-nomadic community.  Conclusions: Unit cost of test-and-treat, and especially among semi-nomadic communities are higher than cost of community-directed treatment with ivermectin. However, it is critical to note that two-years implementation show a significantly larger impact in infection prevalence than the preceding 20 years of annual communitydirected treatment with ivermectin. Implementation might be a cost-effective intervention in regions where prevalence of mf is still high, or in hard-to-reach areas where community-directed treatment with ivermectin has failed to reach a high enough coverage to stop transmission or where marginalised populations consistently miss treatment. |
| Additional Information: |  |
| Question | Response |
| Government Employee  Are you or any of the contributing authors an employee of the United States government?  Manuscripts authored by one or more US Government employees are not copyrighted, but are licensed under a [CC0 Public Domain Dedication,](https://creativecommons.org/about/cc0) which allows unlimited distribution and reuse of the article for any lawful purpose. This is a legal requirement for US Government employees. | No - No authors are employees of the U.S. government. |

| This will be typeset if the manuscript is accepted for publication. |  |
| --- | --- |
| Financial Disclosure  Enter a financial disclosure statement that describes the sources of funding for the work included in this submission and the role the funder(s) played. This includes grants and any commercial funding of the work or authors.  This statement will be typeset if the manuscript is accepted for publication.  Review the [submission guidelines](https://journals.plos.org/plosntds/s/submission-guidelines#loc-financial-disclosure-statement) and the  instructions link below for detailed requirements and guidance. | This work was supported by Sightsavers, using no specific grant. The study was conducted by Sightsavers’ research department and authors working outside Sightsavers did not receive funding for their work related to this article. |
| Competing Interests  On behalf of all authors, disclose any competing interests that could be perceived to bias this work.  This statement will be typeset if the manuscript is accepted for publication.  Review the instructions link below and PLOS NTDs’ [competing interests](https://journals.plos.org/plosntds/s/competing-interests#loc-what-represents-a-competing-interest) policy to determine what information must be disclosed at submission. | The authors have declared that no competing interests exits. |
| Data Availability  Provide a Data Availability Statement in the box below. This statement should detail where the data used in this submission can be accessed. This statement will be typeset if the manuscript is accepted for publication.  Before publication, authors are required to make all data underlying their findings | Financial data used for the analysis are available in supporting information. |

| fully available, without restriction. Review our [PLOS Data Policy](https://journals.plos.org/plosntds/s/data-availability) page for detailed information on this policy. Instructions for writing your Data Availability statement can be accessed via the Instructions link below. |  |
| --- | --- |

Manuscript	[Click here to access/download;Manuscript;Manuscript.docx](https://www.editorialmanager.com/pntd/download.aspx?id=1207984&guid=8b69f43e-6585-4aa3-9d95-505cad93a080&scheme=1)

1 Cost of implementing doxycycline test-
2 and-treat strategy for onchocerciasis
3 elimination among settled and semi-
4 nomadic groups in Cameroon

5

6 **Authors:** Guillaume Trotignon^1^, Ruth Dixon^1^, Kareen Atekem^2^, Laura Senyonjo^1^,
7 Joseph Kamgno^3,4^, Didier Biholong^5^, Iain Jones^1^, Rogers Nditanchou^2^

8

9 ^1^Sightsavers, 35 Perrymount Road, Haywards Heath RH16 3BZ, UK; ^2^Sightsavers, Cameroon Country 10 Office, P.O. Box: 4484 Yaoundé, Cameroon; ^3^Centre de recherche sur la filariose et d’autres maladies 11 tropicales (CRFilMT), Street 1.411, Fouda Quarter, Yaounde, Cameroon; 4 Faculty of Medicine and 12 Biomedical Sciences, University of Yaounde I, ^5^National Programme for the Control of Lymphatic

13	filariasis and Onchocerciasis, Ministry of Public Health, Yaounde, Cameroon

14

15	**Corresponding authors:** Guillaume Trotignon gtrotignon@sightsavers.org

16

# 17 **Authors’ contribution**

18 **Guillaume Trotignon:** Conceptualization, Formal Analysis, Data Curation, Investigation, 19 Methodology, Project Administration, Software, Visualization, Writing – Original Draft 20 Preparation, Writing – Review & Editing

21 **Ruth Dixon**: Conceptualization, Data Curation, Formal Analysis, Funding Acquisition,
22 Methodology, Supervision, Validation, Writing – Review & Editing
23 **Kareen Atekem**: Data Curation, Resources, Writing – Review & Editing
24 **Laura Senyonjo**: Project Administration, Writing – Review & Editing
25 **Joseph Kamgno**: Project Administration, Writing – Review & Editing
26 **Didier Biholong**: Project Administration, Writing – Review & Editing
27 **Iain Jones**: Project Administration, Validation, Resources, Writing – Review & Editing
28 **Rogers Nditanchou**: Conceptualization, Data Curation, Funding Acquisition, Methodology,
29 Project Administration, Resources, Validation, Writing – Review & Editing

30

31

32 **Financial support:** This work was supported by Sightsavers, using no specific grant.
33 The study was conducted by Sightsavers’ research department and authors working 34 outside Sightsavers did not receive funding for their work related to this article.

35

36	**Data sharing:** Financial data used for the analysis are available in supporting 37	information.

38

39	**Conflict of interest:** The authors have declared that no competing interests exits.

40

# Abstract

**Background:** Onchocerciasis is a neglected tropical disease with 217.5 million people globally are at risk of having the infection. Sightsavers carried out test-and-treat with doxycycline in settled and semi-nomadic communities of Massangam Health District in Cameroon. This paper presents the related cost of activity implementation in two community contexts, settled and semi-nomadic.

**Methods:** A mix of gross or micro-costing was used to identify cost components, and bottom-up and top-down approaches for their valuation. The opportunity costs of vehicle and equipment use was estimated and included. However, opportunity costs of building use and Ministry of Public Health staff salaries were not included. We only captured the incremental costs of implementing test and treatment activities as part of a functional annual community-directed treatment with ivermectin programme.

**Results:** We estimate an economic cost per person tested and cost per person treated in Massangam to be US$134 and US$664, respectively. Total implementation cost in the settled community was US$78,965 and US$69,677 in the semi-nomadic community.

**Conclusions:** Unit cost of test-and-treat, and especially among semi-nomadic communities are higher than cost of community-directed treatment with ivermectin. However, it is critical to note that two-years implementation show a significantly larger impact in infection prevalence than the preceding 20 years of annual communitydirected treatment with ivermectin. Implementation might be a cost-effective intervention in regions where prevalence of mf is still high, or in hard-to-reach areas where community-directed treatment with ivermectin has failed to reach a high enough coverage to stop transmission or where marginalised populations consistently miss treatment.

# Author Summary

Onchocerciasis, also called river blindness, is a neglected tropical disease that human contract via bites of the *Simulium* blackflies. These blackflies usually nest along rivers and spread a parasite worm to nearby population. To eliminate and stop the transmission of the parasitic worm *Onchocerca volvulus*, a yearly distribution of medication Ivermectin has been conducted in infected areas. This medication kills the worm’s larvae but not the adult worm, which is however affected by the antibiotic doxycycline. After over 20 years of mass distribution of Ivermectin, some regions still experience transmission of the disease. This is the case of the Massangam Health District in Cameroon. Therefore, alternative strategies have been envisaged to stop transmission. These includes the treatment of nomadic communities specifically, two times a year treatment with Ivermectin and the distribution of positive tested persons with Doxycycline. This paper looks at the cost of implementing these specific alternative strategies to compare with the current elimination strategy to demonstrate the potential effectiveness of these interventions.

# Introduction

Onchocerciasis is a neglected tropical disease (NTD) caused by a parasitic nematode, *Onchocerca volvulus*. It is prevalent in rural and poor settings in Africa and is transmitted to humans through the bites of infected black flies of the genus *Simulium,* that breed in fast flowing rivers and streams. The disease results in stigma and disability due to skin lesions, severe itching, and ocular disorders including visual impairment which can lead to irreversible blindness (1, 2). In 2019, the World Health Organization (WHO) estimated that 217.5 million people globally are at risk of having the infection and that more than 99% of all onchocerciasis cases are in sub-Saharan Africa (3). In the new WHO Roadmap on NTDs 2021-2030, onchocerciasis has been targeted for elimination. There are geographical locations under WHO recommended ivermectin mass drug administration (MDA) that have persistent high transmission or unsatisfactory progress toward elimination of onchocerciasis (4). The WHO has recommended a series of different strategies in these areas to address these challenges and move towards elimination (5, 6).

Massangam Health District (Figure 1) in the West Region of Cameroon is not on track to eliminate onchocerciasis, despite receiving 20 years of uninterrupted annual distribution of ivermectin (7, 8). To redress this situation Sightsavers, in collaboration with the national control programme, experienced test-and-treat with doxycycline (TTd) as part of a suite of alternative treatment strategies (ATS) that also included larviciding and biannual MDA (9). For the TTd component of the ATS, all eligible (age >8 years, not breastfeeding, not pregnant and not severely ill) individuals were screened through skin snip microscopy for onchocercal microfilarial (mf) infection and those positive (presence of mf) were offered 100mg of doxycycline with a light meal, daily for 35 days under directly observed therapy (DOT, daily observation) strategies by trained community drug distributors (CDDs) (9). Two rounds of TTd were conducted among settled communities, the first in July 2017 and the second in September 2018, following a sequence sensitisation, census, screening, and treatment. Another round of TTd targeted,semi-nomadic communities between October and December 2019.

*Figure 1: Map of Massangam Health District showing settled and semi-nomadic settlements (map created using ArcGIS Pro with data collected by Sightsavers).*

***Legend Figure 1****: Map of the Massangam Health District showing settled communities (red squares), occupied semi-nomadic camps (orange dots) and unoccupied camps (green dots) covered during test-and-treat activities. Bottom right corner shows a map of Cameroon with Massangam district highlighted for national context.*

During the first two implementations of ATS, the presence and challenges of reaching semi-nomadic populations with TTd and ivermectin came to light. In response, adaptations to the strategies were developed and implemented, including culturally adapted mobilisation and sensitisation materials - banners, flyers, posters, video, and audio tapes in four local languages (Pidgin English, French, Bamoun and Fulfulde) used at national, regional, district and community levels. In addition, mobilisation and sensitisation were extended and reinforced in every camp by two CDDs – one from the settled community and the other from the semi-nomadic community in a “buddiedup” system. For semi-nomads, daily monitoring was unfeasible, and treatment was monitored daily for the first two days and then weekly, with two additional checks by phone each week.

Within the semi-nomadic communities two further activities were piloted around the TTd with the aim of further increasing coverage. Firstly, the identification of seminomadic settlements by satellite imagery and ground truthing was undertaken to supplement census activities conducted via community networks. Secondly, skin snips found to be negative by microscopy were examined by polymerase chain reaction (PCR) in the Center for Research in Filaria and other Tropical Diseases (CRFilMT) laboratory, Yaounde. This was to identify further positive cases for treatment and to understand the sensitivity of the microscopy in a low-intensity setting.

In this paper we estimate the cost of implementing a TTd programme in two community contexts, settled and semi-nomadic. We have focussed on the second round of TTd that was implemented in July 2018 among the settled communities of Massangam, and between October 2019 and December 2019 among the semi-nomadic population in encampments. We estimate the implementation cost and examine the cost drivers for each scenario.

Secondly, we have costed the two supplementary activities conducted in the seminomadic implementation (satellite camp identification and microscopic negative sample examined by PCR). These are optional activities and are referred to as ‘addons’ throughout this paper. We estimate the gains in terms of coverage of testing and treating positive cases through each add-on.

Finally, we discuss the implications of these costs in decision making for treatment programme selection and implementation. We reflect on the trade-off between coverage and cost for the two add-ons and compare the findings with communitydirected treatment with ivermectin (CDTi), including the impacts on disease prevalence, intensity and timeline to elimination. This analysis provides a foundation which future endeavours to eliminate onchocerciasis can build on.

# Methods

## Costing approach

The interventions were implemented in three high infection communities (Njinjia/Njngouet, Makakoun, Makouopsap) in the Massangam Health District, and jointly implemented by the Ministry of Public Health of Cameroon, Sightsavers and CRFilMT. Different measurement and cost valuation methodologies were used as the level of detail necessary was not obtained to accurately estimate the cost of several activities (see supplementary information Table S1). This study estimates the cost borne by the three service providers (Ministry of Public Health of Cameroon, CRFilMT, and Sightsavers) to conduct one phase of TTd among settled groups and one phase among semi-nomadic groups. Following existing guidelines, a mix of gross or microcosting was used to identify cost components, and bottom-up and top-down approaches for their valuation (10). Standard costs are costs of activities that, based on project experience, are deemed necessary for an optimal implementation. The opportunity costs of vehicle and equipment use was estimated and included. The value of volunteered time of CDDs was evaluated using the opportunity cost approach (11). As pilot activities took place in a rural area, we used the yearly “agriculture, forestry, and fishing, value added per worker” from the World Bank dataset as a shadow wage to value CDDs’ daily work (12). However, opportunity costs of building use and Ministry of Public Health staff salaries were not included. We only captured the incremental costs of implementing TTd activities as part of a functional annual CDTi programme.

## Data collection

Actual expenditure and budgeted expenditure for conducting ATS activities were collected retrospectively from implementing partners and Ministry of Public Health between 2017 and 2020. Sightsavers expenditure were downloaded as a transaction list from the financial tracking system and consolidated via Excel spreadsheets.

## Data analysis

The delivery of TTd includes several activities, some of which were adapted or added to suit the nomadic community context. Details of the activities are given in the table below (Table 1).

188	*Table 1: Test-and-treat standard activities with adaptation and add-on for semi*189	*nomadic groups*

| **Activities** | **Settled communities**  **(September 2018)** | | | **Semi-nomadic communities**  **(October to December 2019)** |
| --- | --- | --- | --- | --- |
| **Planning and coordination** | All activities related to operational, management and coordination of TTd activities. | | | Idem |
| **Advocacy and**  **Sensitisation** | Meeting with national and local stakeholders, sharing information about the TTd programme at the local level. | | | Culturally adapted mobilisation and sensitisation materials (videos and audios in different languages). |
| **Training** | Training of field staff, including settled community CDDs. | | | Training of semi-nomadic CDDs. |
| **Census** | Team goes to community and goes door-to-door registering people and determining eligibility and sensitising to the upcoming test. | | | Team goes to the actual camps after undergoing a community listing process and snowballs at each camp to identify unlisted camp. Then, hut-to-hut registering people. |
| **Testing** | Skin snip and field examination under microscope. | | | Same approach but a nomad community guide accompanies the team. |
| **Treatment and**  **Monitoring** | Another team follows up positive results - conducting pregnancy test where appropriate before treating with DOT with a meal provided before medicine is swallowed. | | | DOT for two days, then a weekly treatment course, selfmonitored in a treatment diary, and funds provided for the meals. Treatment team calls (twice) during the week and visit weekly to monitor treatment. |
| **Satellite**  **nomadic camp**  **identification^a^** | Not implemented communities | in | settled | For nomadic camps, in addition to the community listing of camps and snowballing, potential camps were identified by satellite and visited to verify if they were occupied. |
| **PCR testing of negative skin snips from nomadic**  **participants^a^** | Not implemented communities | in | settled | Due to the low sensitivity of microscopy, negative samples from the semi-nomads were retained and further analysed by PCR. |

190 *^a^Add-ons will be presented separately as they are not standard TTd activities*
191 **Legend**: DOT (doxycycline under direct observation); PCR (Polymerase Chain Re-
192 action)

Project costs were collected or valued then categorised into specific project activities and cost categories to identify cost drivers and unit costs (see supplementary information Table S2). All capital expenditure, equipment with usefulness exceeding one year, were annualised assuming an average life of five years (13).

The total standard cost of implementing a TTd strategy was estimated based on the project output and financial data. The cost by activity and category are presented, with and without add-ons, along with estimates of the cost per person screened and cost per person treated, for both settled and semi-nomadic communities (see supplementary information Dataset S3).

## Currency

As recommended by Turner et, al. all costs were converted to US Dollars of 2020 (USD) from Francs CFA (XAF) or UK pounds (GBP) using the average monthly exchange rate for the year 2020, and adjusted for inflation to USD 2020 using consumer price indexes (14-16).

# Results

The total cost of test-and-treat activities in settled communities was US$78,965 (Table 2). Planning and coordination activities represented the highest share of costs (34% of total cost, US$23,576), followed by treatment activities (26%, US$17,772). Overall, personnel represented the highest cost category (48% of total expenditure) followed by materials and supplies (31%).

215 *Table 2: Cost of test-and-treat activities in settled communities by activities and* 216 *categories (in USD 2020)*

| **Activities/** **Categories** | **Personnel** | **Materials and supplies** | **Transportation** | **Total** |
| --- | --- | --- | --- | --- |
| **Planning and**  **coordination** | 21,205.1 (31%) | 27.0 (0%) | 2,343.6 (3%) | 23,575.6 (34%) |
| **Advocacy and** **sensitisation** | 2,943.4 (4%) | 2,681.1 (4%) | 1,771.7 (3%) | 7,396.2 (11%) |
| **Training** | 551.9 (1%) | 0.0 (0%) | 60.0 (0%) | 611.9 (1%) |
| **Census** | 3,484.8 (5%) | 70.1 (0%) | 1,597.9 (2%) | 5,152.8 (8%) |
| **Testing** | 1,401.6 (2%) | 5,941.1 (9%) | 2,752.8 (4%) | 10,095.5 (15%) |
| **Treatment** | 1,857.2 (3%) | 12,634.7 (18%) | 3,279.8 (5%) | 17,771.6 (26%) |
| **Monitoring of testand-treat** | 1,797.5 (3%) | 0.0 (0%) | 2,264.2 (3%) | 4,061.7 (6%) |
| **Sub-total** | 33,241.5 (48%) | 21,353.9 (31%) | 14,069.9 (21%) | 68,665.4 (100%) |
| **Overheads^a^** | - | - | - | 10,299.8 |
| **Total** | - | - | - | 78,965.2 |

217	*^a^Overheads estimated at 15% of sub-total activity costs*

218

In semi-nomadic communities the cost of the adapted test-and-treat activities was US$69,677.9 (Table 3). In terms of activity, testing was the highest proportion of costs (23% of total cost, US$13,891). A significant share of this (US$7,500) being due to materials and supplies needed for the testing team to camp and conduct the sample collection and examination on site. Personnel represented the highest cost category with 45% of total cost.

Table 3: Cost of test-and-treat activities in semi-nomadic communities by activities and categories (without add-ons, in USD 2020)

| **Activities/ Categories** | **Personnel** | **Materials and supplies** | **Transportation** | **Total** |
| --- | --- | --- | --- | --- |
| **Planning and coordination** | 9,154.4 (15%) | 2,151.5 (4%) | 175.2 (0%) | 11,481.1 (19%) |
| **Advocacy and sensitisation** | 5,431.3 (9%) | 3,781.8 (6%) | 2,084.9 (3%) | 11,298.0 (19%) |
| **Training** | 867.3 (1%) | 447.7 (1%) | 262.8 (0%) | 1,577.7 (3%) |
| **Census** | 4,274.9 (7%) | 1,770.6 (3%) | 3,027.5 (5%) | 9,073.0 (15%) |
| **Testing** | 2,803.3 (5%) | 7,500.0 (12%) | 3,588.2 (6%) | 13,891.4 (23%) |
| **Treatment** | 918.0 (2%) | 1,238.7 (2%) | 1,252.5 (2%) | 3,409.2 (6%) |
| **Monitoring of test-andtreat** | 4,007.9 (7%) | 0 (0%) | 5,851.2 (10%) | 9,859.1 (16%) |
| **Sub-total** | 27,457.0 (45%) | 16,890.2 (28%) | 16,242.3 (27%) | 60,589.5 (100%) |
| **Overheads^a^** | - | - | - | 9,088.4 |
| **Total** | - | - | - | 69,677.9 |

*^a^Overheads estimated at 15% of sub-total activity costs*

The overall economic cost of implementing a TTd with a tailored strategy to reach settled and semi-nomadic groups in Massangam amounted to US$148,643, with a cost per person screened of US$134 and a cost per person treated of US$664 (Table

4).

A cost difference is to be noted when looking separately at the implementation among settled and semi-nomadic communities. TTd strategy cost US$78,965 among the settled communities and cost among semi-nomadic communities was US$69,678. In terms of unit cost per person tested and treated, it corresponds to US$90 and US$387 respectively in settled communities, and US$308 and US$3,484 for semi-nomadic groups without add-ons, and US$392 and US$4,425 with add-on activities.

241	*Table 4: Total cost per community and per outcome (US$ 2020)*

|  | Number of per-  sons censused | Number of persons screened | Number of persons treated | Total economic cost  (including overheads) | Cost per person censused | Cost per person screened | Cost per person treated |
| --- | --- | --- | --- | --- | --- | --- | --- |
| Settled communities | 1,617 | 881 | 204 | 78,965 | 49 | 90 | 387 |
| Semi-nomadic communities | 748 | 226 | 20 | 69,678 | 93 | 308 | 3,484 |
| Total Massangam (without  add-ons) | 2,365 | 1,107 | 224 | 148,642 | 63 | 134 | 664 |

242

243

The add-on activities amounted to US$16,359, increasing the total TTd programme cost by 13% (Table 5). Amounting to US$112 per additional person screened and US$1,365 per person treated. The camp verification allowed to detect two more camps and 11 additional persons, where the PCR tests allowed to detect 14 infections for 161 tests conducted among semi-nomadic camps.

250 *Table 5: Incremental cost and outcomes of add-ons among semi-nomadic*
251 *communities (US$ 2020)*

|  | Additional persons censused | Additional persons screened | Additional persons treated | Total economic cost  (including overheads) | Cost per person censused | Cost per person screened | Cost per person treated |
| --- | --- | --- | --- | --- | --- | --- | --- |
| Camp verification | 11 | 11 | - | 8,931 | 812 | 812 | - |
| PCR analysis | - | 161 | - | 7,429 | - | 46 | - |
| Additional treatment | - | - | 14 | 2,753 | - | - | 197 |
| Total add-ons | 11 | 172 | 14 | 19,113 | 1,738 | 112 | 1,365 |

252 **Legend**: PCR (Polymerase Chain Reaction)

253

# Discussion

This study shows how much alternative treatment strategies would cost in settled communities and semi-nomadic communities in hard-to-reach areas. To the best of our knowledge, this is the first cost estimate of onchocerciasis treatment among seminomadic groups. From an empirical based study, financial and output data, we estimate an economic cost per person tested and cost per person treated in Massangam to be US$134 and US$664, respectively. Total implementation cost in the settled community was US$78,965 and in the semi-nomadic community it was US$69,677. Overall, the total cost of implementing the doxycycline test-and-treat strategy for onchocerciasis elimination in Massangam came to US$167,456.

Unit costs are estimated to be higher among semi-nomadic groups compared to unit costs among settled groups because implementation required more staff and more time, both of which are important cost drivers. Indeed, adapted sensitisation and census activities were more complicated than in settled communities, as camps were not always occupied, and satellite maps needed to be reconciled with field verification visits to complete the census. Teams travelling for census or testing work had to stay several days in the field and conduct laboratory analysis at camps, increasing the number of working days and transportation costs. Finally, in addition to “settled” CDDs, nomad CDDs were employed to accompany field work which further increased personnel costs.

Unit costs in settled communities were also relatively high compared to the economic cost of CDTi, the benchmark for Cameroon ranges from US$2.65 per person (in (adjusted to US$ 2020 using consumer price index) at an implementation scale of 10,000 persons, to US$0.26 for a scale of 1,000,000 persons treated (15, 17). The difference in cost is due to the TTd approach requiring more material and supplies than would otherwise be used for CDTi, and to the pilot scale and innovative nature of the implementation. We would expect in a scaled situation that fixed costs would be reduced by the inclusion of a greater number of communities, and variable costs would be optimised through the reduction of many of the exploratory costs and enhanced monitoring implicit in implementing innovative programming for the first time.

Also, when comparing CDTi to TTd it is critical to note that TTd is curative, rather than targeting a break of transmission as CDTi does. A break of transmission requires up to 15 years of repeated programming, and in many cases where TTd is being considered it is because long term CDTi has not had the required impact. Initial results of an impact assessment for this implementation showed a package of alternative treatment strategies implemented for two years in Massangam (two years of the TTd strategy, biannual CDTi and ground larviciding) had a significantly larger impact on infection prevalence in two years than the preceding 20 years of annual CDTi (Atekem et al, in press).

Given the early stage of the impact survey and the small scale of the pilot, we are not in the position to conclude on the cost-effectiveness of this approach and there is need for further research in this area. TTd implementation might be a cost-effective intervention in regions where prevalence of mf is still high, or in hard-to-reach areas where CDTi has failed to reach a high enough coverage to stop transmission or where marginalised populations consistently miss treatment. There have been reports of transmission being interrupted in certain places, but hard-to-reach foci remain that keep driving transmission (18). In such places, TTd will result in faster progress to elimination in the whole area, instead of endless cycles of MDA. Even if the foci has low intensity infection, transmission will be perpetuated (19). The addition of camp verification and PCR tests will detect individuals with low infection and thus help to drive down infection. Indeed, during the second phase of the pilot, two camps and 11 additional people were found through camp verification and 161 PCR tests were conducted, detecting 14 additional infections in semi-nomadic communities, at an incremental cost of $46 per person screened (Table 5). In addition, in loa loa endemic areas where loascope is being used to exclude people from being treated with ivermectin, TTd should be considered for those being excluded from ivermectin treatment. This highlights how TTd could be an effective tool to address many of the challenges to onchocerciasis elimination.

Despite the rigorous financial data collection, our costing estimates suffer from several limitations. First, there was limited details provided for some of the activities, namely planning and coordination, advocacy, and overheads, which meant costs could not be fully disaggregated and required the use of a mix of costing methodologies (see supplementary information Table S1). In addition, some of the planning and coordination expenditures were shared with previous rounds of implementation of TTd. For instance, the data analyst and coordinator time could not be directly attributed to the studied implementation phase and their full cost have been included which has inflated the presented costs.

The last mile of disease elimination is often the most difficult and the most expensive. Elimination of onchocerciasis will only be achieved if effective interventions are delivered to all the people in need, including the hardest-to-reach. Understanding the costs of different strategies and the costs of targeting different groups, including nomadic populations, is essential for the planning and budgeting of activities to achieve the goal of onchocerciasis elimination in Cameroon and elsewhere.

# Acknowledgement

We would like to thank Richard Selby for his support and comments on the manuscript, Alexandre Chailloux for creating the map of Massangam Health District, the Centre de recherche sur la filariose et autres maladies tropicales (CRFilMT), the National Programme for the Control of Lymphatic filariasis and Onchocerciasis, and Sightsavers Cameroon office for the coordination and implementation of activities. Finally, we acknowledge particularly the communities of Massangam for their cooperation.

# References

1. Gideon Informatics I, Berger S. Onchocerciasis: Global Status. Los Angeles, UNITED STATES: Gideon Informatics, Incorporated; 2021.
2. World Health Organization. Guidelines for stopping mass drug administration and verifying elimination of human onchocerciasis. 2016.
3. Malecela MN, Ducker C. A road map for neglected tropical diseases 2021– 2030. Transactions of The Royal Society of Tropical Medicine and Hygiene. 2021;115(2):121-3.
4. Lakwo T, Oguttu D, Ukety T, Post R, Bakajika D. Onchocerciasis Elimination: Progress and Challenges. Res Rep Trop Med. 2020;11:81-95.
5. Boussinesq M, Fobi G, Kuesel AC. Alternative treatment strategies to accelerate the elimination of onchocerciasis. International Health.

2018;10(suppl_1):i40-i8.

6. World Health Organization & African Programme for Onchocerciasis Control (APOC). Report of the consultative meetings on strategic options and alternative treatment strategies for accelerating onchocerciasis elimination in Africa. African Programme for Onchocerciasis Control.; 2015.
7. Bakajika D, Senyonjo L, Enyong P, Oye J, Biholong B, Elhassan E, et al. Ongoing transmission of human onchocerciasis in the Massangam health district in the West Region of Cameroon: Better understanding transmission dynamics to inform changes in programmatic interventions. PLOS Neglected Tropical Diseases. 2018;12(11):e0006904.
8. Senyonjo L, Oye J, Bakajika D, Biholong B, Tekle A, Boakye D, et al. Factors Associated with Ivermectin Non-Compliance and Its Potential Role in Sustaining Onchocerca volvulus Transmission in the West Region of Cameroon. PLOS Neglected Tropical Diseases. 2016;10(8):e0004905.
9. Atekem K, Dixon R, Wilhelm A, Biholong B, Oye J, Djeunga HN, et al. Evaluating the impact of alternative intervention strategies in accelerating onchocerciasis elimination in an area of persistent transmission in the West Region of Cameroon. medRxiv. In press:2022.06.19.22276627.
10. Hendriks ME, Kundu P, Boers AC, Bolarinwa OA, Te Pas MJ, Akande TM, et al. Step-by-step guideline for disease-specific costing studies in low- and middleincome countries: a mixed methodology. Glob Health Action. 2014;7:23573.
11. Turner HC, Toor J, Bettis AA, Hopkins AD, Kyaw SS, Onwujekwe O, et al. Valuing the Unpaid Contribution of Community Health Volunteers to Mass Drug Administration Programs. Clinical infectious diseases : an official publication of the Infectious Diseases Society of America. 2019;68(9):1588-95.
12. The World Bank. Agriculture, forestry, and fishing, value added per worker (constant 2015 US$). 2022.
13. McFarland D, Menzies N, Njoumemi Z, O O. Study of Cost Per Treatment with Ivermectin using CDTI Strategy. African Programme for Onchocerciasis Control (APOC); 2005.
14. Financial Times. FT Guide to World Currencies. In: Morningstar, editor. 2021.
15. International Monetary Fund. World Economic Outlook Database: April 2021. 2021.
16. Turner HC, Lauer JA, Tran BX, Teerawattananon Y, Jit M. Adjusting for Inflation and Currency Changes Within Health Economic Studies. Value in Health. 2019;22(9):1026-32.
17. Fitzpatrick C, Fleming FM, Madin-Warburton M, Schneider T, Meheus F, Asiedu K, et al. Benchmarking the Cost per Person of Mass Treatment for Selected Neglected Tropical Diseases: An Approach Based on Literature Review and Metaregression with Web-Based Software Application. PLOS Neglected Tropical Diseases. 2016;10(12):e0005037.
18. Katabarwa MN, Habomugisha P, Eyamba A, Byamukama E, Nwane P,

Arinaitwe A, et al. Community-directed interventions are practical and effective in lowresource communities: experience of ivermectin treatment for onchocerciasis control in Cameroon and Uganda, 2004-2010. International health. 2016;8(2):116-23.

19. Basáñez MG, Townson H, Williams JR, Frontado H, Villamizar NJ, Anderson RM. Density-dependent processes in the transmission of human onchocerciasis: relationship between microfilarial intake and mortality of the simuliid vector. Parasitology. 1996;113 ( Pt 4):331-55.

Table S1 – Methodologies by activity and sources used

[Click here to access/download](https://www.editorialmanager.com/pntd/download.aspx?id=1207981&guid=f4fe4be7-a533-44bc-9dac-268cebdb4fac&scheme=1)

[Supporting Information renamed_8b5b8.pdf](https://www.editorialmanager.com/pntd/download.aspx?id=1207981&guid=f4fe4be7-a533-44bc-9dac-268cebdb4fac&scheme=1)

Table S2 - Table of activities included in standard cost

[Click here to access/download](https://www.editorialmanager.com/pntd/download.aspx?id=1207982&guid=2f38398b-6197-4f9d-8733-16233129907b&scheme=1)

[Supporting Information](https://www.editorialmanager.com/pntd/download.aspx?id=1207982&guid=2f38398b-6197-4f9d-8733-16233129907b&scheme=1)

[Table S2 - Table of activities included in standard cost.pdf](https://www.editorialmanager.com/pntd/download.aspx?id=1207982&guid=2f38398b-6197-4f9d-8733-16233129907b&scheme=1)

Dataset S3 - Standard costs

[Click here to access/download Supporting Information](https://www.editorialmanager.com/pntd/download.aspx?id=1207983&guid=edf6773e-1483-421f-b775-93f8201d4827&scheme=1)

[Dataset S3 - Standard costs.xlsx](https://www.editorialmanager.com/pntd/download.aspx?id=1207983&guid=edf6773e-1483-421f-b775-93f8201d4827&scheme=1)
