# Supplementary material for: Cost of implementing a doxycycline test-and-treat strategy for onchocerciasis elimination among settled and semi-nomadic groups in Cameroon
Source: PLoS Negl Trop Dis. 2023 Oct 18;17(10):e0011670. doi: 10.1371/journal.pntd.0011670 (PMC10615284; doi:10.1371/journal.pntd.0011670)
Supplement: Attachment — Submitted filename: Response to reviewers.docx [file pntd.0011670.s005.docx]

Guillaume Trotignon	31.07.2023

35 Perrymount Rd.

Haywards Heath

RH16 3BZ, UK

Dear Editors, dear Reviewers,

On the behalf of all authors, I want to thank you for taking the time to review our manuscript.

We think that the reviews were extremely useful and relevant. We have tried to address all other concerns which were all pertinent to improve the quality of the manuscript or clarify it.

You will find below answers to your comments (answers in blue).

Thank you for taking the time to consider our revised manuscript.

Best regards,

Guillaume Trotignon

Dear Trotignon,

Thank you very much for submitting your manuscript "Cost of implementing doxycycline test-and-treat strategy for onchocerciasis elimination among settled and semi-nomadic groups in Cameroon" for consideration at PLOS Neglected Tropical Diseases. As with all papers reviewed by the journal, your manuscript was reviewed by members of the editorial board and by several independent reviewers. The reviewers appreciated the attention to an important topic. Based on the reviews, we are likely to accept this manuscript for publication, providing that you modify the manuscript according to the review recommendations.


Please prepare and submit your revised manuscript within 30 days. If you anticipate any delay, please let us know the expected resubmission date by replying to this email.

When you are ready to resubmit, please upload the following:

[1] A letter containing a detailed list of your responses to all review comments, and a description of the changes you have made in the manuscript.
Please note while forming your response, if your article is accepted, you may have the opportunity to make the peer review history publicly available. The record will include editor decision letters (with reviews) and your responses to reviewer comments. If eligible, we will contact you to opt in or out

[2] Two versions of the revised manuscript: one with either highlights or tracked changes denoting where the text has been changed; the other a clean version (uploaded as the manuscript file).

Important additional instructions are given below your reviewer comments.

Thank you again for your submission to our journal. We hope that our editorial process has been constructive so far, and we welcome your feedback at any time. Please don't hesitate to contact us if you have any questions or comments.

Sincerely,

Sabine Specht
Academic Editor
PLOS Neglected Tropical Diseases

Nigel Beebe
Section Editor
PLOS Neglected Tropical Diseases

***********************


Reviewer's Responses to Questions

<b>Key Review Criteria Required for Acceptance?</b></br></br>
As you describe the new analyses required for acceptance, please consider the following:</br></br>

<b>Methods</b></br></br>
-Are the objectives of the study clearly articulated with a clear testable hypothesis stated?</br>
-Is the study design appropriate to address the stated objectives?</br>
-Is the population clearly described and appropriate for the hypothesis being tested?</br>
-Is the sample size sufficient to ensure adequate power to address the hypothesis being tested?</br>
-Were correct statistical analysis used to support conclusions?</br>
-Are there concerns about ethical or regulatory requirements being met?</br></br>

Reviewer #1: (No Response)

Reviewer #2: Described below

Reviewer #3: - The objectives are clearly articulated with the hypothesis
- The study design is appropriate to address the stated objectives
- The population is clearly described and appropriate for the hypothesis
- No. The sample size of the semi-nomadic population is too small to ensure adequate power to address question.
- The study did not require elaborate statistical analysis.
- There are no concerns about ethical or regulatory requirements

--------------------

<b>Results</b></br></br>
-Does the analysis presented match the analysis plan?</br>
-Are the results clearly and completely presented?</br>
-Are the figures (Tables, Images) of sufficient quality for clarity?</br></br>

Reviewer #1: (No Response)

Reviewer #2: Described below

Reviewer #3: - Yes. The analysis presented matched the plan
- Results are clearly and completely presented
- The figures are of sufficient quality and are clear

--------------------

<b>Conclusions</b></br></br>
-Are the conclusions supported by the data presented?</br>
-Are the limitations of analysis clearly described?</br>
-Do the authors discuss how these data can be helpful to advance our understanding of the topic under study?</br>
-Is public health relevance addressed?</br></br>

Reviewer #1: (No Response)

Reviewer #2: (No Response)

Reviewer #3: - Yes
- Yes
- Yes, the authors discussed how the outcome will stimulate further larger study
- Public Health relevance not really addressed going by the text unless the researchers took care of it but did not spell it out in the text, for example, precautions taken during the skin snips.

--------------------

<b>Editorial and Data Presentation Modifications?</b></br><br/>
Use this section for editorial suggestions as well as relatively minor modifications of existing data that would enhance clarity. If the only modifications needed are minor and/or editorial, you may wish to recommend “Minor Revision” or “Accept”.

Reviewer #1: (No Response)

Reviewer #2: • Line 57, Abstract: For readers it might be more informative to provide additional detail in the Results section, even if that requires a more succinct Conclusions section.Thank you for your comment. I have added the total economic and financial cost of the strategy to the result section (line 55 to 57).

• Line 62, Abstract: what is ‘mf’?Mf stands for microfilarial. We have replaced the abbreviation for the full name (line 64).

• Lines 77-78: The Author Summary mentions a twice-yearly Ivermectin strategy as being included in the evaluation, but this is not mentioned in the abstract.We have added this information in the abstract, line 42.

• Line 100: ‘experienced’ – is this the wrong word?Change to implemented, which is more accurate (line 102).

• Line 132: It might be useful to define ‘ground truthing’.\ A short definition was added line 169 to 170 plus wording was changed to camp verification for more clarity

• Line 245-246: Seems like there is typo here (sentence fragment)? The following sentence also seems to be off. Thank you, it was indeed unclear. Lines 269 to 272 were amended.

• Line 258: ‘From an empirical based study, financial and output data, we…’ this text is unclear. Line 289, we tried to simplify it.

• Line 276: ‘(in’ Should this text be removed?Indeed, thank you.

• In general: The manuscript could use copy-editing as there were several places where there appeared to be grammar issues or missing words. I have listed some below but there may be others. We agreed. We have sent the manuscript to an independent proof-reader.

Reviewer #3: Yes. These are stated in the body of the text for correction by the authors.

--------------------

<b>Summary and General Comments</b></br></br>
Use this section to provide overall comments, discuss strengths/weaknesses of the study, novelty, significance, general execution and scholarship. You may also include additional comments for the author, including concerns about dual publication, research ethics, or publication ethics. If requesting major revision, please articulate the new experiments that are needed.

Reviewer #1: This paper quantifies the cost of implementing a doxycycline test and-treat strategy for onchocerciasis elimination among settled and semi-nomadic groups in Cameroon. This is a novel and well presented costing study on an important area.

My only major comment is that the perspective of the analysis needs to be explicitly stated in the methods (i.e. the costs were estimated under the perspective of _______). On a related note, the fact that opportunity costs of building use and Ministry of Public Health staff salaries were not included needs to be clearly justified in the methods in some way and this point expanded on in the limitations.

Thank you for your review and comments. We have added details on the perspective lines 199 to 202 and on the opportunity costs lines 188 to 196 and lines 350 to 353. We hope it clarifies and justifies the approach used.

I also believe that it would be helpful if the difference between estimated total financial and economic costs was stated.

Thank you for the suggestion. We have added financial costs information in table 4 and 5 and amended the text accordingly (line 263 and line 277)

Please state what discount rate was used for the annualization.

We have changed the annualization factor by adding a 3% discount rate as recommended in the WHO-CHOICE guidelines. Please see lines 210 to 212.

Reviewer #2: This manuscript describes the costs of intensified onchocerciasis control activities in rural Cameroon, where doxycycline and more frequent ivermectin distribution was used. The costing is fairly straight-forward and in general the methods seem appropriate (though note some questions/suggestions below). The writing is generally good but there are occasionally dropped words or sections where the meaning is unclear, so a careful readd-through would be helpful to resolve these.

MAJOR ISSUES

• Line 100: The Introduction section of the manuscript contains detailed information about the intervention that I would normally expect to see in the Methods Section (e.g. under an ‘Intervention and Setting’ subheading). The authors could consider shifting this detail to the Methods section, though this may be a matter of preference / journal style.

Thank you. A setting section has been added where the detailed interventions has been moved (see Methods section).

• Line 155 (Methods): The abstract mentions that MOH staff salaries are not included, yet I cannot find this information in the Methods section. This would be important to describe here along with the activities they perform that are excluded from the costing.

Yes, we appreciate that is an important limit to the study. Although we tried to obtain more detailed information on opportunity costs, we failed to obtain enough information to estimate it. It was mainly due to the time lapse between implementation of project activities and the actual collection of economic information. See changes lines 188 to 196 and lines 350 to 353.

• Line 155 (Methods): It would be useful to include in the methods a clear statement of the study time frame (i.e. the period of activities being evaluated). There are several dates given in the Introduction, then different dates in line 181, so it is unclear to me right now.

Yes, indeed. I have modified line 107 and 145 to 148. Table 1 has been moved and modified to include dates and “round” excluded from the costing study. We hope it clarifies the study frame.

• Line 195-196: Capital equipment was annualized assuming a useful life of 5 years. Conventionally these calculations include a discount rate representing the opportunity cost of capital investment. Was this done? If so the discount rate should be included here (commonly 3% or 5%).

Indeed, the annualization was done for accounting purpose but in the context of the cost-outcome study and to comply with existing guidelines (WHO-CHOICE) we have used a 3% discount rate and modify the manuscript accordingly (see lines 210 to 212).

• Line 203: This information on discounting does not seem quite right, or may need to be edited for clarity. Ideally the costs would first be inflated to current values in local currency units, then converted to USD at current market exchange rates.

We followed the Turner et al. first adjustment method, as most of the equipment were imported tradable resources and personnel staff costs did not evolve between 2019 and 2020. We have added justification line 222 to 224.

• Other comments: It would be helpful to review the CHEERS 2022 reporting framework ([https://www.equator-network.org/reporting-guidelines/cheers/](https://protect-eu.mimecast.com/s/rYZsCElPyfW7rVXhx5OEO?domain=equator-network.org)) to make sure all relevant items are reported. This reporting framework was built for cost-effectiveness analyses and so there will be parts of it that are not needed for a cost analysis, but still good to use.

Thank you for your suggestions. We have followed the “Step-by step guideline for disease-specific costing studies in low-and middle-income countries: a mixed methodology” from Hendriks et al. (2014).

• The manuscript could use copy-editing as there were several places where there appeared to be grammar issues or missing words. I have listed some below but there may be others.

We have sent the manuscript to a copyeditor to correct grammar issues and typos (See various changes throughout the manuscript).

Reviewer #3: (No Response)

--------------------

PLOS authors have the option to publish the peer review history of their article (<a href="[https://journals.plos.org/plosntds/s/editorial-and-peer-review-process#loc-peer-review-history](https://protect-eu.mimecast.com/s/twq9CGZ9ASJ7xKXTr8f6L?domain=journals.plos.org)" target="_blank">what does this mean?</a>). If published, this will include your full peer review and any attached files.<br><br>

If you choose “no”, your identity will remain anonymous but your review may still be made public.<br><br>

<b>Do you want your identity to be public for this peer review?</b> For information about this choice, including consent withdrawal, please see our <a href="[https://www.plos.org/privacy-policy](https://protect-eu.mimecast.com/s/yPVhCJZP1S8JG3MiKthya?domain=plos.org)" target="_blank">Privacy Policy</a>.

Reviewer #1: No

Reviewer #2: Yes: Nicolas A Menzies

Reviewer #3: No


Figure Files:
While revising your submission, please upload your figure files to the Preflight Analysis and Conversion Engine (PACE) digital diagnostic tool, [https://pacev2.apexcovantage.com](https://protect-eu.mimecast.com/s/r8VBCKO6GTqogzpsk0046?domain=pacev2.apexcovantage.com). PACE helps ensure that figures meet PLOS requirements. To use PACE, you must first register as a user. Then, login and navigate to the UPLOAD tab, where you will find detailed instructions on how to use the tool. If you encounter any issues or have any questions when using PACE, please email us at [figures@plos.org](mailto:figures@plos.org).

Data Requirements:
Please note that, as a condition of publication, PLOS' data policy requires that you make available all data used to draw the conclusions outlined in your manuscript. Data must be deposited in an appropriate repository, included within the body of the manuscript, or uploaded as supporting information. This includes all numerical values that were used to generate graphs, histograms etc.. For an example see here: [http://www.plosbiology.org/article/info:doi/10.1371/journal.pbio.1001908#s5](https://protect-eu.mimecast.com/s/icBqCLgPGsPWxy8uYvjXZ?domain=plosbiology.org).

Reproducibility:
To enhance the reproducibility of your results, we recommend that you deposit your laboratory protocols in protocols.io, where a protocol can be assigned its own identifier (DOI) such that it can be cited independently in the future. Additionally, PLOS ONE offers an option to publish peer-reviewed clinical study protocols. Read more information on sharing protocols at [https://plos.org/protocols?utm_medium=editorial-email&utm_source=authorletters&utm_campaign=protocols](https://protect-eu.mimecast.com/s/yMCRCMj9Xf5rKMmFq0g0q?domain=plos.org)

References
Please review your reference list to ensure that it is complete and correct. If you have cited papers that have been retracted, please include the rationale for doing so in the manuscript text, or remove these references and replace them with relevant current references. Any changes to the reference list should be mentioned in the rebuttal letter that accompanies your revised manuscript. If you need to cite a retracted article, indicate the article's retracted status in the References list and also include a citation and full reference for the retraction notice.

__________________________________________________
In compliance with data protection regulations, you may request that we remove your personal registration details at any time. (Use the following URL: [https://www.editorialmanager.com/pntd/login.asp?a=r](https://protect-eu.mimecast.com/s/5sOMCNx8GUNWKDys3qLxf?domain=editorialmanager.com)). Please contact the publication office if you have any questions.
